# A mechanistic computational model of the HIF signaling pathway in endothelial cells

**DOI:** 10.1016/j.isci.2026.116195

**Published:** 2026-06-02

**Authors:** Rebeca Hannah de Melo Oliveira, Arvind P. Pathak, Aleksander S. Popel

**Affiliations:** 1 Department of Biomedical Engineering, The Johns Hopkins University School of Medicine, Baltimore, MD, USA; 2 Russell H. Morgan Department of Radiology and Radiological Sciences, The Johns Hopkins University School of Medicine, Baltimore, MD, USA; 3 Sidney Kimmel Comprehensive Cancer Center, The Johns Hopkins University School of Medicine, Baltimore, MD, USA

**Keywords:** Computational bioinformatics, Molecular biology, Systems biology

## Abstract

In conditions such as cancer, cardiovascular diseases, and retinal diseases, cells under hypoxia activate oxygen-sensing mechanisms, promoting adaptation and survival. Many hypoxia computational models predate standardized identifiability analyses and lack systematic treatment of the HIF isoform-specific dynamics in endothelial cells. We present a technically validated mechanistic model of the HIF pathway in endothelial cells, capturing graded oxygen sensitivity and the transition from HIF1α-dominated acute to HIF2α-dominated prolonged hypoxic responses. Following identifiability analyses, the model was calibrated and validated against independent datasets, achieving Pearson correlations of 0.7–0.95 and no systematic residual bias (Runs test *p* ≥ 0.35). Simulations revealed dose-dependent HIF stabilization and *VEGFA* mRNA induction, a time-dependent shift in transcriptional control from HIF1*α* to HIF2*α*, and non-redundant isoform-specific effects of PHD2 and PHD3 inhibition. This validated model provides a robust mechanistic framework for studying endothelial hypoxia signaling, suitable for integration into larger computational models of ischemic disease.

## Highlights

• Integrative mechanistic model of the HIF1 to HIF2 switch in endothelial cells under hypoxia
• PHD2 and PHD3 play distinct isoform-specific roles in regulating hypoxia-inducible factors
• Model predicts fractional HIF1 and HIF2 contributions to VEGFA during prolonged hypoxia

## Graphical abstract

**Figure:**
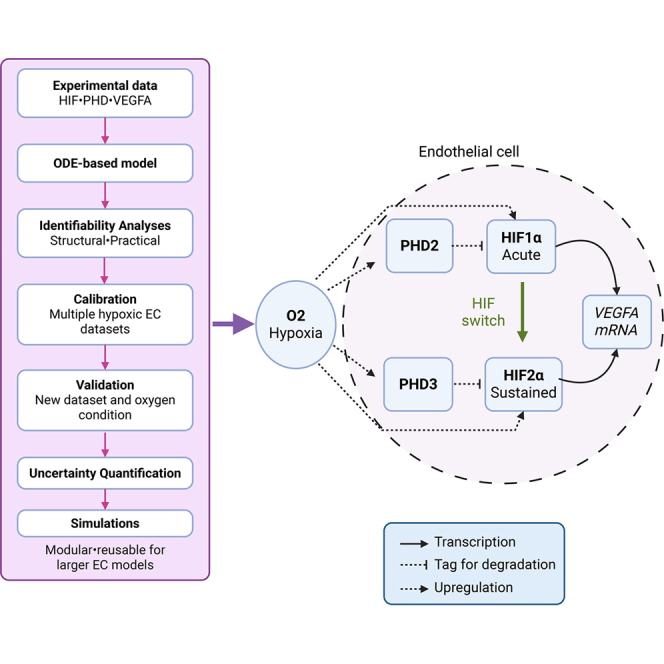

## Introduction

Oxygen is among the key players in life, maintaining intracellular ATP levels to provide energy for different biological processes and taking part in a multitude of biochemical reactions. Under different pathological scenarios, a decrease in oxygen levels can result in a condition termed hypoxia.^1^ Among hypoxic scenarios, the most studied are those in solid tumors, cardiovascular diseases (such as peripheral arterial disease, or PAD), and retinal diseases (such as neovascular [wet] age-related macular degeneration).^2^^,^^3^ Endothelial cells (ECs), which line all blood vessels in the body, serve as conduits for blood flow and facilitate nutrient and oxygen exchange. Restricted blood flow (i.e., ischemia) is an important driver of tissue hypoxia. To maintain homeostasis under hypoxia, cells activate oxygen-sensing mechanisms that trigger adaptive responses aimed at restoring normoxia and promoting cell survival.^4^

The mechanism through which cells sense hypoxia and respond to it has been thoroughly investigated over the past several decades. A family of heterodimeric transcription factors, hypoxia-inducible factors (HIFs), activates genes that regulate glycolysis, cell cycle, angiogenesis, and cell survival.^5^^,^^6^ For instance, ECs in ischemic tissues stabilize HIF1*α* and HIF2*α*, which translocate to the nucleus, dimerize with constitutively expressed HIF1*β*, and lead to the transcription of rescue factors, such as vascular endothelial growth factor (*VEGFA*). *VEGFA* triggers sprouting angiogenesis toward hypoxic regions, thus supplying oxygen and re-establishing the normal environment.^1^ Both HIF1 and HIF2 influence endogenous *VEGFA* levels in ECs, and their inhibition under hypoxia downregulates *VEGFA* levels.^7^^,^^8^^,^^9^ Previous studies have highlighted the differential effects of HIF1 and HIF2 on regulating angiogenesis through modulation of endothelial cell proliferation, migration, adhesion, survival, and vascular density.^10^ These non-linear, context-dependent interactions are difficult to study experimentally, but mathematical models can systematically capture their dynamics and predict responses under varied experimental conditions.

**Figure 1:**
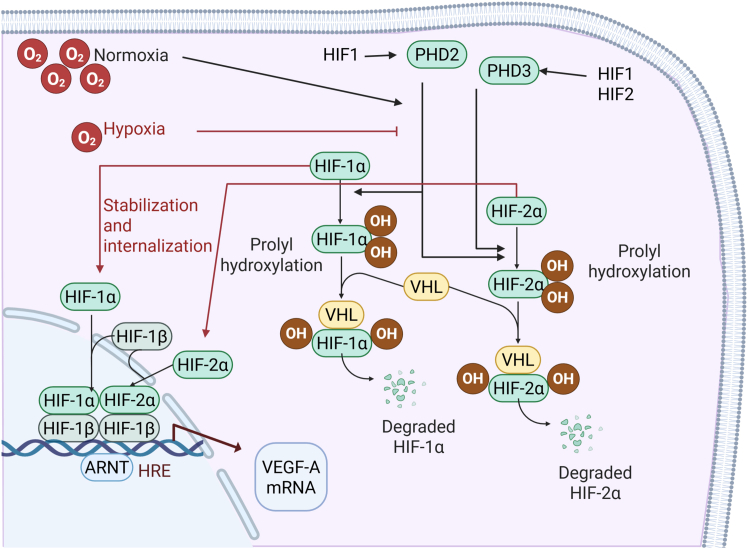
Diagram of the HIF signaling pathway and the upregulation of VEGF Under normoxia, PHDs mark HIF*α* for degradation mediated by VHL. Under hypoxia, PHDs’ hydroxylation of HIF1*α* is reduced, and HIFs are stabilized and internalized. In the nucleus, HIF1*α* dimerizes with HIF1*β* and interacts with HREs, promoting the transcription of growth factors, such as *VEGF* mRNA, to stimulate angiogenesis and rescue homeostasis. Diagram was created with BioRender.com.

HIFs exist in three known forms in humans: HIF1α, HIF2α, and HIF3α, all of which are sensitive to oxygen levels variation. The *β* subunit (HIF1*β*), on the other hand, is constitutively expressed and not responsive to oxygen levels. HIF regulation depends on oxygen sensitive enzymes called prolyl hydroxylases (PHDs). Under normoxia, PHDs hydroxylate HIF*α*, which leads to HIF recruitment by von Hippel-Lindau (vHL) proteins and subsequent ubiquitination and proteasomal degradation.^11^ Under hypoxia, reduced PHDs’ activity allows HIF stabilization and transcriptional activation of angiogenic factors such as *VEGFA*. This process is depicted in Figure 1. HIF1 and HIF2 can act either synergistically (e.g., enhancing the expression of VEGF and erythropoietin) or antagonistically (e.g., HIF1*α* enhancing the expression of nitric oxide synthase gene and HIF2*α* regulating arginase 1 expression, which has opposing effects on macrophage polarization).^12^ HIF3*α* is a less studied form of HIFs, considered an inhibitory element due to its lack of a transactivation domain.^13^ This complex regulation drives the need for computational models of the HIF-PHD-VHL interactions to predict the HIF response under different oxygen levels.

Studies comparing PHD isoforms (PHD1, PHD2, and PHD3) and their activity on oxygen-sensing and HIF degradation have provided insights into how they affect and are affected by HIF*α* levels.^11^^,^^14^ PHD2 and PHD3 predominate in ECs, with HIF1*α* promoting both, and HIF2*α* promoting only PHD3. Although PHD1 has an effect on HIF regulation, it is a less studied form in the context of ECs, and previous studies indicate that its effect is smaller than that of the other PHDs,^14^^,^^15^ but with an effect on regulating the activity of nuclear factor kappa B (NF-kB).^16^ By modeling these complex feedback loops between HIFs and PHDs, we can better understand, simulate and observe how perturbations affect the molecular-level responses of ECs.

In this context, mechanistic computational models enable us to investigate the effects of hypoxia on various signaling pathways and predict the effects of therapeutic approaches aimed at restoring homeostasis. Although mechanistic models have been developed to elucidate the oxygen sensing mechanism and the molecular response that follows hypoxia,^11^^,^^17^^,^^18^^,^^19^^,^^20^^,^^21^^,^^22^^,^^23^ many of these do not systematically include the roles of HIF1 and HIF2 in ECs, or often lack major modeling steps such as rigorous identifiability analysis and uncertainty quantification. Moreover, these models often lack validation against new publicly available experimental datasets.

**Figure 2:**
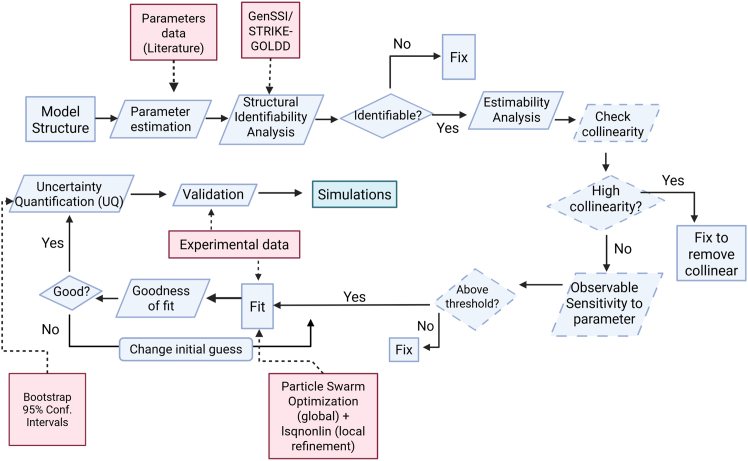
Methodology for model building, calibration, and validation Pink boxes connected to the main modeling steps by dashed arrows represent data used as inputs to the model and specific methods employed in this work. Blue boxes represent the major steps in the workflow. The final step (simulations) is highlighted in a dark blue box, since it is only performed after passing all delineated requirements. Blue boxes with dashed borders describe sub-steps within the practical identifiability analysis step.

**Figure 3:**
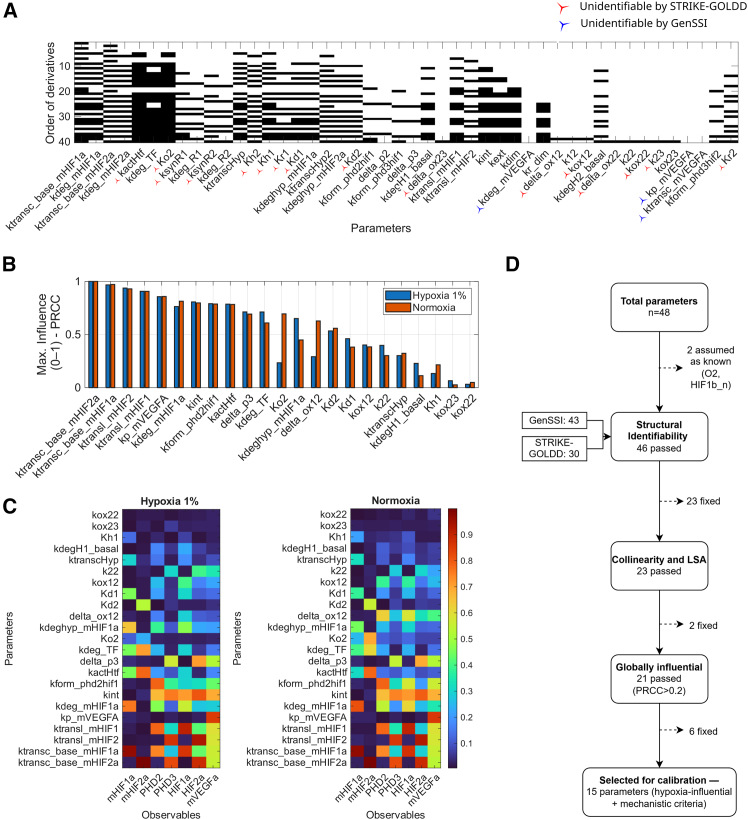
Identifiability analyses indicate 21 identifiable parameters (A) Identifiability tableau showing structurally identifiable parameters obtained with GenSSI2.0. The time derivative order evaluated is indicated on the *y* axis and the parameter name on the *x* axis. Blue markers show parameters found as unidentifiable by GenSSI, and red markers show parameters marked as unidentifiable by STRIKE-GOLDD. (B) Combined global sensitivity analysis (PRCC) of the 23 non-collinear structurally identifiable parameters under hypoxia (1% O_2_) and normoxia (21% O_2_), showing the most influential parameters overall. (C) Global sensitivity analysis under normoxia (21% O_2_) and hypoxia (1% O_2_), based on PRCC absolute value of the 23 non-collinear parameters showing their influential effect on each observable. Values are normalized to their maximum over 48 h. (D) Summary of parameter selection process to parameters to calibrate with available data.

Therefore, the proposed model will include major mechanisms described in recent literature, such as the HIF1/2 transition.^11^ Also, in the present model we will incorporate modern methodologies, such as structural and practical identifiability analysis, and uncertainty quantification metrics,^24^^,^^25^^,^^26^ which ensure model robustness and fidelity (Figures 2 and 3). The result is a minimal, focused mechanistic model of the PHD/HIF/*VEGFA* axis in ECs, calibrated and validated against human umbilical vein endothelial cells (HUVEC)-specific experimental data, designed to serve as a modular component for integration into larger computational models of hypoxia-induced angiogenesis and disease.

## Results

### Model recapitulates trends and data used for calibration and validation

**Figure 4:**
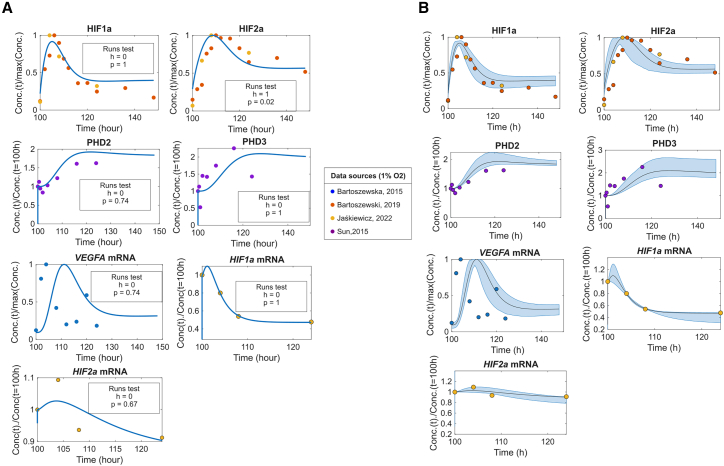
Calibrated model reproduces experimental data trends (A) Time-course simulations of model observables compared to experimental measurements used for calibration. Colored markers represent data extracted from multiple literature sources.^11^^,^^18^^,^^27^^,^^28^ Simulations were performed under hypoxic conditions (1% O_2_). Prior to hypoxia exposure, the system was simulated to steady state under normoxia (t = 100 h), after which oxygen levels were reduced to 1% O_2_. To align experimental and simulated timelines, experimental time points were shifted such that their baseline corresponds to the simulated normoxic steady state (t = 100 h). Model fits are shown as solid lines. (B) Model predictions with uncertainty bounds obtained from residual bootstrapping (1,000 replicates). Shaded regions represent the 95% prediction confidence intervals computed from the empirical distribution of simulated outputs. Experimental data are overlaid for comparison. For each observable, residual randomness was assessed using the runs test, with reported values of h (test decision) and *p* (*p* value).

**Figure 5:**
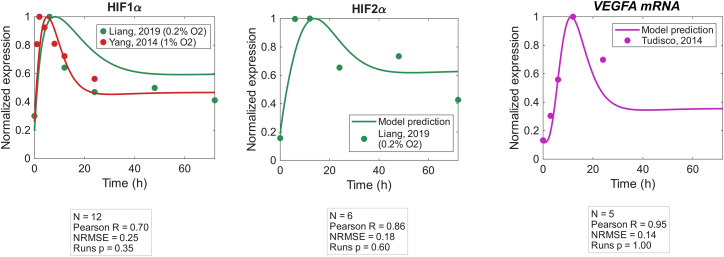
Model reproduces HIF1α, HIF2α protein, and *VEGFA* mRNA dynamics in HUVECs under hypoxia Model predictions (lines) are compared against published time-course data (points) from independent datasets.^29^^,^^30^^,^^31^ Goodness-of-fit metrics (Pearson R, NRMSE, and runs test *p* value) are shown for each species.

Having defined the set of identifiable parameters to fit, we performed global fitting using particle swarm optimization (PSO) to estimate the values of the unknown identifiable parameters. The fitted model responses compared with experimental data are presented in Figure 4A. Overall, the simulated responses closely recapitulated the experimental trends across all observables. Runs tests indicated that residuals were randomly distributed for 6 out of 7 observables, supporting the absence of systematic bias in the model fits. Following calibration, we then evaluated prediction uncertainty through 95% confidence intervals and subsequently validated the model against a new set of data not used during fitting (Figure 5).

Our simulations showed that the computational model captures the overall dynamics of the system. Following global optimization with PSO, we performed local refinement using lsqnonlin on the most influential parameters to improve convergence. Uncertainty quantification was performed on the locally refined solution, as this represents the final calibrated parameter set used for all subsequent simulations. Approximately 50% of experimental data points fell within the 95% confidence intervals (Figure 4B), specifically, 10/15 for HIF1*α*, 7/16 for HIF2*α*, 4/8 for PHD2, 2/8 for PHD3, 2/8 for *VEGFA* mRNA, and 4/4 for both HIF1*α* and HIF2*α* mRNAs. While bootstrap confidence intervals computed on the pre-refinement PSO solution yielded broader bands with approximately 66% point coverage, these reflect the less-converged global solution and are not reported as the primary uncertainty estimate for consistency with the final model parameterization. While this may appear lower than expected, it reflects the integration of heterogeneous datasets collected across different studies, experimental conditions, and measurement platforms, rather than limitations in the model structure.

The largest deviations were observed for PHD2, PHD3, and the *VEGFA* mRNA. Importantly, despite these deviations, the model consistently reproduced the temporal trends and relative dynamics of these species. For the *VEGFA* mRNA specifically, data from Bartoszewska (2015)^27^ suggested a biphasic response with two peaks over the 24 h hypoxic period. While the calibrated model did not reproduce both peaks, it successfully captured the early peak following hypoxia onset, which represents the dominant and most reproducible feature across datasets.^27^^,^^30^^,^^32^ Furthermore, model predictions remain consistent with the independent validation dataset (Figure 5), supporting the robustness of the underlying regulatory mechanisms.

Validation against independent datasets supported the calibrated model’s ability to reproduce the behaviors of HIF1α and HIF2α proteins and the *VEGFA* mRNA under hypoxia (Figure 5). Pearson correlations ranged from 0.70 to 0.95 across validation observables, with the lowest agreement observed for HIF1α, where the validation dataset was collected under 0.2% O_2_ rather than the 1% O_2_ condition (to which the model was originally calibrated). Runs test confirmed that prediction errors were randomly distributed in all cases (h = 0, *p* ≥ 0.35), reinforcing that the model generalized beyond the calibrated data without systematic bias.

### Model captures oxygen-level sensitivity and responds to hypoxia-reoxygenation simulations

**Figure 6:**
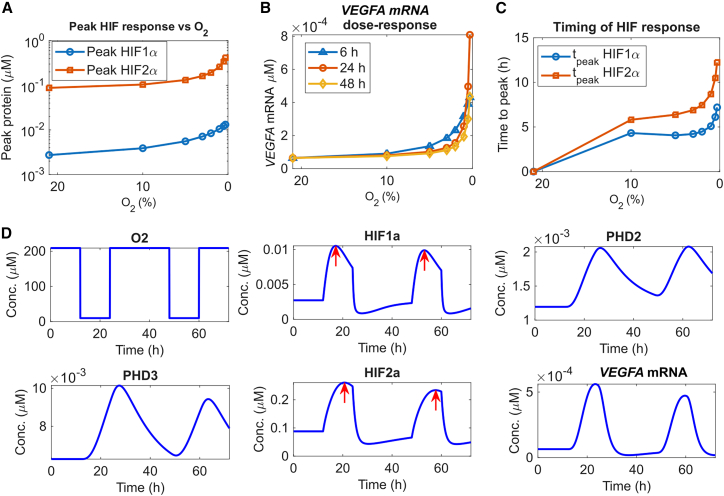
Model predicts oxygen-dependent HIF responses and dynamics under hypoxia-reoxygenation (A) Peak concentrations of HIF1α and HIF2α obtained from simulations at different oxygen levels (O_2_, %). (B) Simulated *VEGFA* mRNA levels at 6, 24, and 48 h across oxygen conditions. (C) Time to peak of HIF1α and HIF2α as a function of oxygen concentration. (D) Temporal profiles of O_2_, HIF1α, HIF2α, PHD2, PHD3, and *VEGFA* mRNA under cyclic hypoxia-reoxygenation. Oxygen levels were varied using step changes between normoxia and hypoxia over the simulation period. Red arrows indicate the time points corresponding to peak HIF1α and HIF2α levels during hypoxic intervals.

To evaluate the model’s response to oxygen availability and its ability to reproduce key hypoxia-driven behaviors, we performed simulations across a range of oxygen levels and under cyclic hypoxia-reoxygenation conditions (Figure 6).

The model predicted a strong increase in peak HIF1*α* and HIF2*α* protein levels (Figure 6A), with HIF2*α* reaching higher steady-state amplitudes. In parallel, the *VEGFA* mRNA showed a dose-dependent increase, with stronger responses at later time points (Figure 6B), reflecting the accumulation of transcriptional outputs under sustained hypoxia. Observing the timing of HIF proteins responses, the model predicted a distinct behavior between isoforms, with HIF1*α* reaching its peak rapidly after hypoxia onset, whereas HIF2*α* showed a delayed peak that occured later and persisted longer, particularly at lower oxygen levels (Figure 6C). This temporal separation agrees with previous experimental reports on the HIF1-to-HIF2 switch under hypoxia.^11^

Under cyclic hypoxia-reoxygenation conditions (Figure 6D), the model indicated that following hypoxia onset at 0 h of simulation, there was an increase in HIF1 and HIF2 protein levels, followed closely by an increase in *VEGFA* mRNA. Re-establishing normoxia at 12 h promptly caused a downregulation of HIF levels and of *VEGFA* mRNA. Another induction of hypoxia at 24 h upregulated HIF1, HIF2, and *VEGFA* mRNA, and re-oxygenation at 48 h once again downregulated HIFs and the *VEGFA* mRNA (Figure 6A). Each hypoxic episode led to rapid stabilization of HIF1 followed by a delayed and more sustained increase in HIF2, accompanied by induction of *VEGFA* mRNA. The hypoxic events are also noticeable on PHD2 and PHD3 behaviors, contributing to feedback regulation and shaping of amplitude and timing of the hypoxic responses. Overall, our simulation indicated the model’s responsiveness to oxygen levels, satisfying the first of our requirements.

Evaluating the peak responses of HIF1 and HIF2 under hypoxia, marked with red arrows in Figure 6D, we can also see that while HIF1 showed an acute response to hypoxia onset, HIF2 showed a later peak response, which lasted longer at higher values than HIF1’s response. This recapitulates the behavior shown in Figure 6C, and satisfies our second requirement of reproducing the HIF switch seen experimentally. Overall, our established model, built with a strict methodological rigor, reproduced experimental behavior seen in ECs, while also incorporating the requirements previously described. These hypoxia-reoxygenation simulations represent testable model predictions. Direct experimental validation could be achieved by exposing HUVECs to cyclic hypoxia using a programmable hypoxia chamber, with western blot quantification of HIF1α, HIF2α, PHD2, and PHD3 protein levels at defined time points during each hypoxic and reoxygenation interval.

To further evaluate the functional consequences of this temporal HIF switch, we quantified the relative contributions of HIF1 and HIF2 to *VEGFA* mRNA production across different time points following hypoxia onset (Figure S2). Early after hypoxia induction (6 h), *VEGFA* mRNA expression was more sensitive to HIF1 inhibition, indicating that HIF1 primarily drives the initial transcriptional response. At intermediate time points (24 h), both HIF1 and HIF2 contributed to *VEGFA* mRNA regulation, with a shift toward increased HIF2 influence. At later time points (48 h), *VEGFA* mRNA expression became predominantly dependent on HIF2 activity.

Additionally, we comparatively explored the model responses to conventional *in vitro* transition (21%–1% O_2_) and physioxic transition (5%–0.5% O_2_) transition, each initialized at their respective normoxic (21% or 5% O_2_) steady states (Figure S4). HIF1α dynamics were largely conserved between conditions, with a modest elevation observed at later time points (beyond ∼24 h) under the physioxic transition, reflecting more complete PHD suppression at 0.5% O_2_. HIF2α stabilization and *VEGFA* mRNA induction were quantitatively larger under the physioxic transition, driven by greater PHD3 suppression and the resulting amplification of late HIF2α-dependent transcriptional output. PHD2 and PHD3 levels were correspondingly elevated during hypoxia under physioxic conditions, consistent with stronger HIF-driven transcriptional feedback. All species returned to their respective normoxic steady-state values upon reoxygenation. As these simulations involve oxygen levels below the calibrated range, experimental validation in HUVECs under sub-1% O_2_ conditions would be required to confirm these predictions.

These results demonstrate that the model not only reproduces the temporal dynamics of HIF stabilization but also captures the transition in transcriptional control of *VEGFA* mRNA from HIF1 to HIF2, consistent with experimental observations of a functional HIF switch.

### PHD activity inhibition differentially regulates HIF1α and HIF2α dynamics

**Figure 7:**
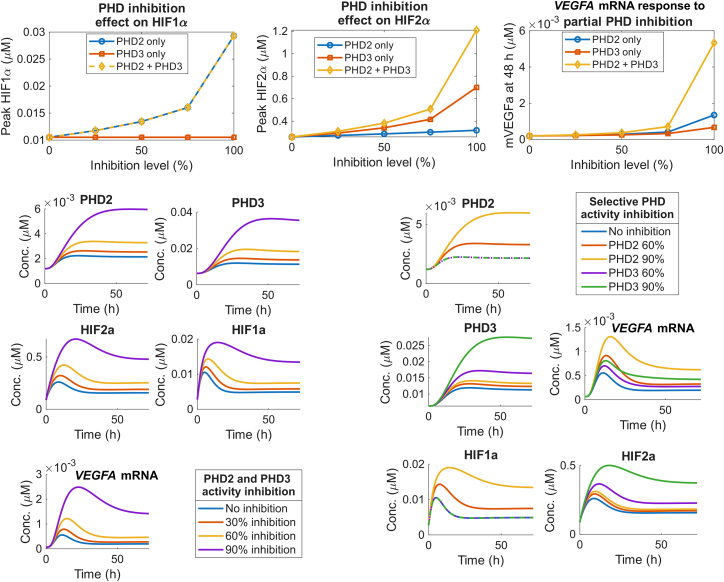
PHD activity inhibition differentially regulates HIF signaling dynamics (A and B) Peak levels of HIF1α (A) and HIF2α (B) as a function of increasing PHD activity inhibition (0%–100%) under hypoxic conditions. (C) *VEGFA* mRNA levels at 48 h in response to partial PHD inhibition, showing a nonlinear increase that is most pronounced under combined PHD2/3 inhibition. (D) Temporal dynamics of PHD2, PHD3, HIF1α, HIF2α, and *VEGFA* mRNA under global (combined) PHD2 and PHD3 inhibition at different levels (0%, 30%, 60%, and 90%). (E) Temporal dynamics under selective inhibition of PHD2 or PHD3 (60% and 90%). Colors denote different inhibition conditions; where curves overlap, distinct line styles are used for clarity.

To investigate the regulatory roles of PHD2 and PHD3 in modulating HIF signaling, we simulated the effects of graded and selective inhibition of PHD activity under hypoxic conditions (Figure 7).

Increasing levels of PHD activity inhibition produced distinct, isoform-specific effects on HIF stabilization. Inhibition of PHD2 activity alone led to an increase in HIF1*α* protein, with less impact on HIF2*α*, whereas PHD3 activity inhibition mostly enhanced HIF2*α* protein accumulation (Figures 7A and 7B), with no significant effect on HIF1*α*. Combined inhibition of both PHD isoforms resulted in a markedly amplified response, indicating a synergistic effect on HIF stabilization, specially HIF2*α*. These effects propagated downstream, with *VEGFA* mRNA expression showing a non-linear increase under PHD inhibition, most evident under dual inhibition scenario (Figure 7C).

Observing how global (dual) or selective activity inhibition affects the temporal dynamics of HIF proteins, *VEGFA* mRNA, and PHDs, the model indicates that global PHD2/3 activity inhibition produces a coordinated, dose-dependent amplification of both HIF1*α* and HIF2*α* responses, characterized by higher peak levels and sustained elevation over time (Figure 7D). This is accompanied by an increase in *VEGFA* mRNA expression and an upregulation of PHD2 and PHD3, reflecting activation of feedback mechanisms within the pathway. Increasing levels of inhibition (30%–90%) progressively increases both magnitude and duration of the hypoxic response.

In contrast, the model predictions indicate that selective inhibition of PHD activities reveals distinct isoform-specific regulatory effects (Figure 7E). Inhibition of PHD2 activity enhances HIF1*α* dynamics, leading to an early increase in HIF1*α* and *VEGFA* mRNA, with comparatively modest effects on HIF2*α*. Conversely, PHD3 activity inhibition predominantly affects HIF2*α* levels, with a weaker effect on HIF1*α*. These differences propagate downstream, resulting in distinct *VEGFA* mRNA expression profiles depending on which PHD isoform activity is inhibited. Together, the results demonstrate that while global PHD inhibition can amplify the overall response to hypoxia, selective inhibition highlights non-redundant and time-dependent roles of PHD2 and PHD3 in HIF signaling dynamics.

To further explore the model’s utility in a pharmacologically relevant context, we simulated cyclic PHD inhibition under normoxic conditions (21% O_2_), mimicking the clinical scenario of PHD inhibitor administration for conditions such as anemia of chronic kidney disease (Figure S3). The results show that PHD inhibition under normoxia produces dose-dependent and isoform-specific HIF stabilization and *VEGFA* mRNA induction qualitatively consistent with the hypoxia simulations, with both HIF isoforms and *VEGFA* mRNA returning to baseline upon inhibitor withdrawal. Notably, a transient undershoot in HIF levels was observed following inhibitor removal, driven by the compensatory upregulation of PHD2 and PHD3 accumulated during the inhibition period. This behavior, which is more pronounced at higher inhibition levels, represents a testable prediction with potential implications for PHD inhibitor dosing schedules. These simulations are presented as model predictions and require experimental validation.

## Discussion

Targeting the HIF pathway for pharmacological intervention has been the focus of several clinical trials, as recently reviewed in a work by Yuan et al.^12^ For example, HIF inhibitors and activators can be used effectively for inducing or suppressing angiogenesis under pathological scenarios, although side effects such as hypoxia and anemia have been reported and require mitigation.^33^^,^^34^ Pathological conditions characterized by hypoxia, such as PAD, are associated with a dysfunctional microvascular network, which prevents restoration of normal oxygen conditions in the ischemic limb.^35^^,^^36^ Moreover, the HIF pathway is also a therapeutic target in multiple cancers^37^^,^^38^^,^^39^ and plays a major role in cardiovascular remodeling, with HIF modulation evaluated in renal anemia^40^ and ischemic heart disease.^5^^,^^41^ In particular, several studies have focused on inhibiting PHDs to activate HIFs as a treatment for ischemic conditions, as recently reviewed by Sousa Fialho et al.^42^

Therefore, with an increasing interest in targeting the HIF pathway and elucidating its mechanisms in various diseases, it is critical to understand the mechanistic aspects of this pathway to design more effective interventions. This motivates the development of computational models for data integration, mechanistic hypothesis testing, and for predicting the effects of therapeutic interventions at the molecular level.

Previous works have investigated the HIF pathway as a therapeutic target, providing insights into cellular adaptation to hypoxia and angiogenesis. For instance, a recent work presented the first model of cerebrovascular endothelial signaling using logic-based differential equations, and it predicted potential therapeutic benefits from inhibiting HIF1α as a treatment for glioma.^43^ Several mechanistic computational models of the HIF pathway have also been reported and reviewed,^19^^,^^21^^,^^44^^,^^45^^,^^46^ advancing our understanding of cellular- and molecular-level responses to hypoxia. For instance, Nguyen et al. (2013) combined *in silico* modeling with *in vitro* experiments to study HIF1*α* stability,^21^ later extended by Fábián et al. to include more downstream signaling.^44^ However, these earlier models lacked structural or practical identifiability analysis and relied on limited calibration datasets, reducing confidence in parameter estimation and predictive capability.

In this work, we developed a minimal, identifiable mechanistic model of the PHD/HIF/*VEGFA* axis in ECs, built on previous modeling work and following rigorous good modeling practices.^47^ This included essential steps such as structural and practical identifiability analysis, systemic calibration, uncertainty quantification, and model validation. Consequently, the resulting model incorporated key components of hypoxia sensing and VEGF regulation through the HIF pathway in ECs, making it suitable for integration into larger mechanistic models of hypoxia-induced angiogenesis and even more complex quantitative systems pharmacology (QSP) models of different diseases, such as discussed by Zhang et al.^48^

Our simulation results indicated that our model was able to capture both the response to various oxygen levels, and the HIF1-HIF2 switch reported experimentally.^39^^,^^49^ Additionally, we evaluated its predictions of HIF proteins and *VEGFA* mRNA responses under hypoxia following activity inhibition of PHD2 and/or PHD3, observing distinct isoform-specific and even synergistic effects (in the case of HIF2*α* and *VEGFA* mRNA expression), consistent with the non-redundant and complimentary roles of the two isoforms. PHD2 inhibition preferentially modulated HIF1α dynamics, whereas PHD3 inhibition had a stronger impact on HIF2α, while dual inhibition resulted in amplified responses across both isoforms and downstream *VEGFA* expression. Our results are consistent with previous reports of PHD2-dominant regulation of HIF1α and PHD3-preferential regulation of HIF2α, demonstrated in ECs: Takeda and Fong (2007) demonstrated that siRNA-mediated PHD2 knockdown enhanced HIF1*α* and *VEGFA* mRNA expression under hypoxia, and Loinard et al. (2009) showed that selective shRNA silencing of PHD3 had a comparatively stronger effect on HIF2*α*-dependent targets^50^^,^^51^ and also in other cell types.^14^^,^^52^ These findings highlight the complementary and non-redundant roles of PHD2 and PHD3 in regulating hypoxia signaling and demonstrate the model’s ability to mechanistically resolve their contributions to HIF dynamics, as well as its potential to test isoform-specific regulatory mechanisms that may be challenging to isolate experimentally. Furthermore, simulations of cyclic PHD inhibition under normoxic conditions (Figure S3) demonstrate that the model can be used to mechanistically test pharmacologically-relevant normoxic scenarios, such as PHD inhibitors for conditions like anemia of chronic kidney disease.^53^ While the results provided in this Figure S3 requires experimental validation, they predict dose-dependent and isoform-specific HIF stabilization consistent with the mechanism of action of clinically used PHD inhibitors.^12^ Similarly, the hypoxia-reoxygenation simulations (Figure 6D) represent testable predictions whose experimental corroboration, for example, through cyclic hypoxia chamber experiments with western blot quantification of HIF and PHD protein levels at defined reoxygenation time points in HUVECs, would be an important next step in establishing the model’s predictive validity beyond the calibrated conditions.

Furthermore, our supplementary simulations comparing conventional (21%–1%) and physioxic (5%–0.5%) oxygen transitions (Figure S4) suggest that while HIF1α dynamics are largely conserved between conditions, HIF2α stabilization and *VEGFA* mRNA induction are modestly but consistently higher under the physioxic transition, reflecting greater PHD3 suppression at the lower oxygen baseline. This demonstrates the importance of the normoxic reference condition selection in quantitatively affecting the model predictions, particularly for HIF2α-dependent outputs, and its relevance to designing *in vitro* hypoxia experiments to reflect physiological oxygen conditions. As these simulations involve oxygen levels below the calibrated range, experimental validation in HUVECs under sub-1% O_2_ conditions would be required to confirm these predictions.

One assumption of our model structure design is the multiplicative formulation used to model *VEGFA* mRNA transcription, which assumes that both HIF1 and HIF2 are jointly needed for full transcriptional activation. While supported by the evidence of synergistic and non-redundant HIF isoform activity at the VEGF promoter,^54^ this finding was demonstrated in Hep3B hepatoma cells. Contradicting evidence exists in other cell types, including in breast cancer cell lines,^55^^,^^56^ and earlier reports on ECs found no HIF2*α* contribution to VEGF secretion in HUVECs.^55^ However, more recent work on HUVECs under sustained hypoxia showed that knockdown of either HIF1*α* or HIF2*α* alone significantly attenuated VEGF production, with HIF2α knockdown being at least as effective as HIF1α knockdown.^32^ This supports our multiplicative formulation in an EC context. Mathematically, the multiplicative structure behaves as an AND gate, potentially underestimating *VEGFA* mRNA in contexts where one isoform dominates. An additive formulation with isoform-specific weights would be an alternative, however, constraining those weights in ECs would require specific knockdown data not currently available, to our knowledge. This would introduce another source of unidentifiable parameters to the model. Future optimization of the model could focus on calibration to such data once available, allowing a formal comparison between the multiplicative and additive formulations.

### Limitations of the study

While our calibration and validation steps, together with goodness-of-fit evaluation via the Runs tests indicated good overall agreement with the experimental data, some discrepancies were observed. In particular, the non-random distribution of HIF2α protein residuals suggests that the mismatch is structured, rather than purely stochastic, pointing to limitations on the current representation of HIF2*α* regulation. This pattern indicates that the model may not fully capture the precise temporal evolution of HIF2*α*, such as timing and shape differences in response. However, when evaluated against the validation dataset, the model reproduced overall amplitude and trend of HIF2*α* dynamics with high accuracy, with minor deviations primarily at the initial time point. This suggests that the observed non-randomness could reflect localized model limitations rather than a failure to capture the dominant system behavior. Additionally, discrepancies in the *VEGFA* mRNA fit and corresponding confidence intervals may be attributed to the presence of a bimodal (two-peak) pattern in the calibration dataset. While the model does not reproduce this double-peak behavior, it accurately captures the overall timing and magnitude of *VEGFA* mRNA induction under hypoxia. This bimodal response, to our knowledge, has not been consistently reported across independent studies in ECs, suggesting that it may reflect dataset-specific variability rather than a robust biological feature. Validating the model on an independent dataset for *VEGFA* mRNA supported this, with the model predicting a peak within the correct temporal window, according to the dataset. Therefore, the model captures the dominant regulatory behavior of *VEGFA* mRNA expression, despite not reproducing the secondary peak observed in the calibration data. It is worth noting that an alternative HIF2*α* validation dataset previously considered was excluded from the revised manuscript,^32^ as its reported dynamics—showing a sustained monotonic increase in HIF2α protein without a subsequent decline—were inconsistent with the behavior observed across multiple independent HUVEC datasets under comparable hypoxic conditions, including those used in the current validation set. This discrepancy may reflect differences in experimental conditions, antibody sensitivity, or the duration and severity of hypoxic exposure across studies, and highlights the inherent variability in quantitative HIF protein measurements across laboratories. Additionally, while the model captures the overall trends in PHD2 and PHD3 dynamics under hypoxia, the confidence intervals do fully encompass all experimental data points. This likely reflects the limited availability and variability of quantitative data for PHD regulation, which constrains parameter estimation and uncertainty characterization. Incorporating additional experimental measurements could improve calibration of these observables and refine the model’s predictive confidence.

It should be noted that model calibration employed a mixed normalization strategy: HIF1α and HIF2α protein levels and *VEGFA* mRNA were normalized to their maximum value within the simulation window, while PHD2, PHD3, *HIF1α* mRNA, and *HIF2α* mRNA were normalized to the initial normoxic steady-state value. This approach preserves fold-change information relative to baseline for species with a meaningful normoxic reference, but it does not allow direct comparison of absolute amplitudes across datasets collected under different experimental conditions and measurement platforms. Therefore, conclusions regarding model performance are limited to temporal dynamics and relative magnitudes of the responses within each dataset. Additionally, we highlight that the data used for model calibration and validation was collected from multiple independent studies using different measurement platforms, and potentially different experimental conditions. Our criteria, given the issue of data limitation, was rather generic, looking for temporal data for each species, collected from ECs (if possible, HUVECs only), under hypoxia conditions. This heterogeneity introduces variability that normalization alone cannot fully account for, and likely contributes to the approximately 50% data coverage within the 95% confidence intervals observed for the calibration results. Despite this, the qualitative temporal features of the HIF response—including the early HIF1α peak and the delayed, more sustained HIF2α response—are consistent across all datasets, supporting the robustness of the model’s main conclusions regardless of cross-study quantitative variability.

An additional model assumption was the generic regulator structures included to represent the temporal changes in *HIF1α* mRNA and *HIF2α* mRNA reported experimentally. Those were included to improve the fitting results of the mRNA, but not as a focus of this work. Future studies evaluating potential regulatory mechanisms of *HIF* mRNAs under hypoxia can replace the generic regulators by specific ones, such as the transcriptional activators NF-κB and Sp1 (which upregulate *HIF1α* mRNA at the promoter level); positive HIF autoregulation via HREs in the HIF1*α* promoter as positive regulators;^57^^,^^58^ and miR-429, miR-155, the ARE-binding protein tristetraprolin (TTP), and the antisense lncRNA HIF1A-AS as negative regulators.^9^^,^^27^^,^^59^^,^^60^^,^^61^

Another limitation of our model included a potential oversimplification of the structures involved in the control of HIF molecules, as included in previous works.^23^ For instance, factor inhibiting HIF1 (FIH) provides an additional oxygen-sensing mechanism but with a weaker effect than PHD2, mainly influencing metabolic adaptation.^12^^,^^62^^,^^63^ A detailed comparative review of these mechanisms has been recently presented and discussed.^4^ Also, during the sensing of O_2_, other mechanisms have been described and modeled, including the interaction of iron (Fe) and 2-oxoglutarate (DG) with PHDs and FIH, prior to interaction with HIFs.^21^^,^^22^^,^^23^ However, our simplified approach allowed us to perform and comply with structural and practical identifiability analysis, which also aided model interpretability. Additionally, while our proposed HIF model was theoretically generalizable to ECs from different tissues, adaptations and additional calibration steps may be required to ensure physiological relevance and model completeness. While our model captures oxygen-dependent PHD regulation, PHD activity is also modulated by metabolic factors including succinate and fumarate, which can competitively inhibit PHD activity and stabilize HIF independently of oxygen levels in disease contexts such as SDH-mutant tumors.^64^ In such settings, our model would be expected to underestimate HIF stabilization, and incorporating these additional regulators is noted as a direction for future development.

While this model provides a validated mechanistic framework for the PHD/HIF/*VEGFA* axis in HUVECs, direct application to *in vivo* disease settings requires caution, as additional factors not captured here—including oscillating oxygen delivery, inflammatory paracrine signaling, and cell-cell interactions—could substantially alter the amplitude and timing of HIF responses. Integration with models of tissue oxygen transport and vascular dynamics would be needed before applying the model predictively to specific disease contexts.

Future developments of the model presented here could incorporate additional regulators of HIFα stability and transcriptional activity, including FIH-1-mediated asparagyl hydroxylation—which controls HIF transactivation independently of proteasomal degradation^65^^,^^66^ —and upstream signaling nodes such as PI3K/Akt and mTORC1 that modulate HIF1α translation and stability.^67^ Also, future model implementation linking *VEGFA* to glycolytic reprogramming targets (e.g., GLUT1 and LDHA) would enable a more accurate mechanistic representation of the HIF-driven metabolic adaptation of ECs to hypoxia.^68^^,^^69^ Also, extending the model to include additional HIF target genes would be a natural and important direction. Finally, experimental validation of the results predicted by this work in hypoxia-reoxygenation and PHD inhibition scenarios would be valuable.

In summary, we present a minimal mechanistic model of the PHD/HIF/*VEGFA* axis in ECs, developed and validated following best practices for systems biology models. By adapting previously published models, we defined and implemented the main aspects that an HIF model should include for increased physiological accuracy. Our final model formulation captures the essential features of oxygen sensing in HUVECs, including graded HIF stabilization across oxygen levels, the isoform-specific HIF1-to-HIF2 switch during sustained hypoxia, and downstream *VEGFA* mRNA regulation, within a compact, identifiable framework. As a validated modular component, it is suitable for integration into larger mechanistic models of hypoxia-induced angiogenesis and quantitative systems pharmacology frameworks targeting hypoxia-related diseases.

## Resource availability

### Lead contact

Requests for further information and resources should be directed to and will be fulfilled by the lead contact Rebeca Hannah de Melo Oliveira (rdemelo1@jhmi.edu).

### Materials availability

This study did not generate new unique reagents.

### Data and code availability

• The authors confirm that the data supporting the findings of this study are available within the article and the supplemental information.
• The computational model developed in this study is publicly available as a Zenodo file (Zenodo: https://doi.org/10.5281/zenodo.19501562) in SBML.
• The datasets employed in this work for model calibration are available in each referred publication and can be found in the original papers. A summary and comparison of the datasets used is included in Table S8.
• All other related items related to the model (i.e., observables, SIA, PIA, reactions, ODEs, species, and parameters) are available in supplemental information (Tables S1–S7) and excel files (Tables S8 and S9). Any further data requests should be directed to the corresponding author.

## Acknowledgments

This work was supported by 10.13039/100000002NIH grant nos. 5R01CA196701, 5R01CA237597, 5R01EY028996, 5R01CA138264, and 5R01HL101200.

## Author contributions

R.H.d.M.O. and A.S.P. developed the concept of the study; R.H.d.M.O. constructed the model, performed all simulations, and wrote a draft of the manuscript; A.P.P. and A.S.P. edited the manuscript.

## Declaration of interests

The authors declare no competing interests.

## STAR★Methods

### Key resources table

**Table:** 

REAGENT or RESOURCE	SOURCE	IDENTIFIER
**Software and algorithms**		

Simbiology	MathWorks	MathWorks, R2026a
ImageJ	NIH	RRID:SCR_003070
SBML implementation of the HIF signaling model and associated dataset	This paper (Data S1); [Zenodo]:	https://doi.org/10.5281/zenodo.19501562
GenSSI 2.0	Ligon et al.^70^	https://doi.org/10.1093/bioinformatics/btx735
STRIKE-GOLDD 4.0	Díaz-Seoane et al.^71^	https://doi.org/10.1093/bioinformatics/btac748

### Method details

#### Key requirements for modeling the HIF pathway

Based on current biological knowledge regarding the oxygen sensing pathway (HIF pathway), we found that at least two elements need to be included in a comprehensive model.

First, the model should be sensitive to the oxygenation level. This means that it should be able to show a dose response to different levels of oxygen used as input. This allows different hypoxia/reoxygenation scenarios to be represented. This also implies that the dynamic response of state variables representing HIFs should show accumulation starting at the onset of hypoxia.

Second, the model should be able to reproduce the switch between HIF1α and HIF2α seen for acute or chronic responses to hypoxia, respectively.^11^^,^^49^ This established mechanism has been recently explored through *in vitro* and in silico models, and reiterates the importance of including PHDs interaction with HIFs and *HIF* mRNA stability.^11^

#### Overall modeling strategy

Based on good modeling practices for building systems biology models, accepted by the systems biology community,^72^ we recently proposed a structured methodology for model design, calibration and validation, including identifiability analyses and uncertainty quantification.^26^ This method improves reproducibility and model trustworthiness and is our standard for performing a technical comparison of methodologies employed in previous works. During the modeling of biological systems, although more detailed models may provide more biological accuracy, the inclusion of intermediate mechanisms is often unnecessary to investigate a designated question. Additionally, excessive complexity can lead to poor modeling and increased uncertainty and unidentifiability issues, especially when experimental data are limited. As the goal of this study is to formulate a new model that provides a platform for simulating oxygen sensing in cells, we evaluated prior models with meaningful differences in terms of their complexity or biological interpretability.

The model described in this work is an ODE-based mechanistic systems biology model calibrated and tested on temporal data from species involved in oxygen sensing through the HIF signaling pathway in ECs.

In this section, we provide a detailed description of the methods used for model development, calibration, validation, and sensitivity analysis of each of the models selected. Figure 2 shows the diagram of our methods, following the previous method presented by our group.^24^^,^^26^ In summary, after defining the research question to be answered via a model, we built the model structure to include the relevant species and parameters. Subsequently, we searched the literature for biologically-accurate values for species’ initial concentrations and parameter values or physiological ranges. Often the values of parameters were not available in the literature, in which case they were deemed to be unknown. One way to find the values of those parameters is applying an inverse modeling strategy, where we optimize the model to find parameter values that reduce the residuals between experimental observations and model predictions. However, several studies have highlighted the importance of performing structural and practical identifiability analyses on the model before performing model optimization. Therefore, we first performed structural identifiability analyses of the model, considering the list of unknown parameters, using well-established toolboxes compatible with Matlab: GenSSI 2.0 and STRIKE-GOLDD 4.0.^70^^,^^71^ Structural identifiability allows us to investigate if the proposed model structure can lead to unique predictions for estimated parameter values. Employing two different methods allows us to find overlapping or distinct parameters classified as structurally identifiable. Thus, we obtained a set of structurally identifiable parameters and structurally unidentifiable parameters. The latter had their values fixed and were considered model assumptions. The former were then evaluated with respect to their practical identifiability, meaning our ability to uniquely identify parameter values based on the experimental data available for the model calibration. To evaluate practical identifiability, we performed two analyses: collinearity, to avoid cancelation of effects on collinear parameters; and sensitivity to the experimental data, to ensure that our estimated parameters influence the data that will be used for model calibration. Parameters that did not pass either of the criteria were estimated or considered as practically unidentifiable. Those that were found to be non-collinear and influential were considered practically identifiable and were then optimized.

Several algorithms are available for model optimization, and we choose a global optimization method, Particle Swarm Optimization (PSO) due to its applicability when dealing with a large parameter space and its availability in SimBiology. We calibrated the model parameters with PSO and a sequential refinement of the most influential parameters using local optimization with lsqnonlin, and we assessed the goodness of fit using a Runs test for randomness on the residuals of the optimization. Next, we performed uncertainty quantification using bootstrap 95% confidence intervals on model predictions, and compared the experimental data used for calibration with the confidence intervals for the model. Finally, we performed model validation on new data that was not used in model calibration and compared the calibrated model predictions to the experimental data. After performing this workflow, we consider our model to be validated, and we could then perform simulations with it.

#### Model formulation

To design and develop our new model of the HIF signaling pathway, we applied the techniques discussed in the STAR Methods section and focused on the reactions essential for complying with the minimum requirements previously discussed. As our intent was to build a model that could be integrated into larger models related to hypoxia-induced angiogenesis, which can significantly increase the level of complexity of the final system, we limited the reactions to include only those consistent with our requirements.

A brief comparison of technical aspects of published models of the HIF pathway^11^^,^^20^^,^^23^ is provided in Table S1 (main species included), Table S2 (model complexity) and Table S3 (technical methodology). Details provided in the tables focus on species, parameters and reactions directly involved in the HIF pathway included in each of the works. Overall, these three studies cover the essential aspects of modeling hypoxia in angiogenesis and their design was based on previous models available in the literature.^19^^,^^21^^,^^22^ Evaluating the structure selection, physiological aspects and methodological approach followed in each of these works, we can identify what to include in a comprehensive new model of the oxygen sensing pathway in ECs to ensure physiological and technical accuracy.

With that in mind, we designed our final model using the Matlab (R2026a) SimBiology toolbox,^73^ including the main HIF*α* isoforms reported in the literature (HIF1*α* and HIF2*α*), their mRNA and protein representations, two PHDs (PHD2 and PHD3) which regulate HIF*α* guided by oxygen availability, HIF1*β*, and *VEGFA* mRNA. As oxygen levels are significantly greater than the initial concentration of PHDs, the reactions are limited mostly by PHDs. Thus, instead of including O_2_ as a state variable, we included it as a parameter in the reactions leading to HIF*α* PHD-dependent degradation. We assumed that HIF1*α* and HIF2*α* were internalized and dimerize with HIF1*β* at the same rate. The transcription of *VEGFA* mRNA was modeled as a multiplicative function of HIF1α and HIF2α activity, where each isoform contributes through a saturable Michaelis–Menten-type term. This formulation encodes the assumption that both isoforms are required for full *VEGFA* mRNA transcriptional activation, consistent with their synergistic and non-redundant roles reported in previous works.^32^^,^^54^ The implications and limitations of this formulation are discussed in the discussion section. Except for HIF1*β*, concentrations of all other species were initially set to zero, and run to steady state after model calibration, and prior to simulations. To represent transcriptional regulation of *HIF1/2α* mRNAs under hypoxia without explicitly modeling all upstream signaling intermediates, we introduced a set of coarse-grained regulatory variables: a generic hypoxia-responsive transcription factor (HypoxiaTF), a repressor of mHIF1α expression (Rep_mHIF1a), and a regulator of mHIF2α expression (Reg_mHIF2a). HypoxiaTF was modeled as an oxygen-sensitive activator whose accumulation increases as oxygen levels decrease, thereby representing the net transcriptional drive induced by hypoxia. Rep_mHIF1a and Reg_mHIF2a were included as abstract controllers downstream of HypoxiaTF to capture delayed and isoform-specific regulation of *HIF* mRNA expression. In this framework, HypoxiaTF promotes hypoxia-induced transcription of both mHIF1α and mHIF2α through saturable terms, while Rep_mHIF1a and Reg_mHIF2a modulate these responses through inhibitory regulatory functions. This formulation was adopted to reproduce the experimentally observed transient and differential dynamics of HIF1α- and HIF2α-related transcripts while keeping the model structurally compact.

In total, our proposed model was composed of 15 species, 47 parameters (including O_2_), 31 reactions, and was described by 14 ordinary differential equations (ODEs). HIF1β was assumed constant, since it is constitutively produced and stable, without oxygen sensitivity.^18^ The initial values for parameters were defined based on ranges available in the literature. The final list of parameters and their pre-fitting values are summarized in Table S4. The full set of ODEs and model reactions are included in Tables S5 and S6. The fitted parameters’ values are presented in Table S7. The initial values of the parameters were set according to the reported in previous studies, when available, and hand-tuned prior to fitting, when not available. HIF half-life is oxygen-dependent, a well-established mechanism for which the Nobel Prize in Physiology or Medicine was awarded in 2019, which we modeled using two degradation rates.^18^ We considered that the basal degradation rate (slow clearance component) of HIF1 and HIF2 proteins was on the order of 10^-5^s^-1^.^20^^,^^74^ Meanwhile, we fit the degradation terms that were oxygen-dependent, after selection through identifiability analyses. For simulations, all time units were converted to common units (i.e. hours and micromoles) for consistency.

For calibration, we obtained time-course data under hypoxia for *HIF1α* mRNA, HIF1*α* (protein), HIF2*α* (protein), EPAS1 (*HIF2α* mRNA) and *VEGFA* mRNA.^11^^,^^18^^,^^27^ We additionally incorporated published data describing PHD2 and PHD3 responses to hypoxia in endothelial cells (HUVECs).^28^ Calibration and validation datasets sources are summarized in Table S8, including oxygen levels, cell type and measurement differences.

#### Structural identifiability analysis (SIA)

After establishing the model structure and initial parameter values, we identified the unknown parameters. Prior to estimating their values, it was necessary to check our ability to determine their values based on the model structure and the available data. This ensured the development of a robust model.^75^^,^^76^ We first performed SIA to determine if the model structure allowed us to infer the internal state of the system based on observables (observability) and if a unique parameterization for any model observable could be obtained (structural identifiability). To ensure a robust evaluation, we used different tools for performing SIA, including GenSSI and STRIKE-GOLDD.^70^^,^^71^^,^^75^ To be considered structurally identifiable, the parameter had to be marked as identifiable by either GenSSI or STRIKE-GOLDD. From the initial set of unknowns, we obtained a subset of structurally identifiable parameters for which we performed estimability analysis. Unidentifiable parameters had their values estimated or were excluded from fitting. Since cell-type and environmental conditions can influence reaction rates, we performed our initial identifiability analysis on all model parameters, except for [O_2_], which was calculated for normoxic and hypoxic conditions, as previously described. Since in our model we consider HIF1*β* as constant, while it is considered as a parameter when setting up the model on GenSSI and STRIKE-GOLDD, it is also considered as known and excluded from the SIA. Parameters without values reported for HUVECs were also evaluated in the identifiability analyses. Therefore, we performed SIA using GenSSI 2.0 and STRIKE-GOLDD 4.0 on 46 of the initial 48 parameters (including HIF1*β*).

The results from GenSSI are shown in the identifiability tableau in Figure 3A, using 8 Lie derivatives. With STRIKE-GOLDD 4.0 *Prob-Obs-Test*,^71^ we evaluated the SIA and observability of the model. GenSSI results showed that almost all parameters evaluated were at least locally structurally identifiable (LSI) Parameters related to *VEGFA* mRNA transcription and degradation were marked as unidentifiable by GenSSI. STRIKE-GOLDD results indicated that all model’s states except for Rep_mHIF1a, Reg_mHIF2a and HypoxiaTF were identifiable, and all parameters but 16 (as shown in Figure 3A) were at least LSI. Since the combined set of structurally identifiable parameters identified by GenSSI and STRIKE-GOLDD indicated that all 46 parameters were at least LSI, we then proceed to evaluate whether the unknown parameters were practically identifiable with all 46 SI parameters.

#### Practical identifiability/estimability analysis

Based on SIA outcomes, we selected the structurally identifiable unknown parameters and performed an estimability analysis to examine if available data were sufficient to uniquely estimate their values. In this work, we consider the estimability analysis equivalent to the PIA evaluation of how data availability limits parameter estimation. The analysis followed a parameter identifiability assessment framework combining local sensitivity analysis and collinearity evaluation (compensation effects of changing a parameter value on the effect of another parameter on the system output).^77^^,^^78^^,^^79^

We first performed local sensitivity analysis using Simbiology, with full normalization of sensitivities, considering experimentally observable species (*HIFs* mRNAs and proteins, PHD2, PHD3 and *VEGFA* mRNA) as outputs (observables). Simulations were conducted under normoxia (21% O2) and hypoxia (1% O2) conditions. For each parameter, the maximum absolute sensitivity across time and outputs was computed, and parameters with low influence (maximum normalized sensitivity <0.01) were classified as non-influential and excluded from further analysis.

To assess parameter redundancy, we constructed a unified sensitivity matrix by combining sensitivity profiles across all conditions and outputs. Collinearity between parameters was evaluated using cosine similarity of their sensitivity vectors, which quantifies similarity in the direction of model response to parameter perturbations. Parameters with cosine similarity greater than 0.99 were considered highly collinear, indicating that their effects on model outputs were not distinguishable. These parameters were grouped into clusters using graph-based connectivity analysis, and for each cluster, only the parameter with the highest influence was retained. Hierarchical clustering of parameter sensitivities was also performed, and the resulting dendrogram is provided in the supplemental information. The dendrogram provides a qualitative visualization of parameter grouping based on similarity of sensitivity profiles, supporting the cluster assignments derived from cosine similarity analysis.

Model observables were defined based on available experimental measurements (e.g., western blot or densitometry data). To enable comparison across datasets, simulated outputs were normalized either to their initial value or to their maximum value within a predefined time window (24, 48, or 72 h) under hypoxic conditions.

Our final set of non-collinear SI parameters consisted of 23 parameters (full list available in the Table S9). Finally, we performed PRCC^80^ for the set of observables in our model HIF1A, HIF2A, *VEGFA* mRNA, *HIF1a* mRNA, *HIF2a* mRNA, PHD3 and PHD2, to check if the 23 parameters selected after excluding collinear factors were overall influential, an indication of practical identifiability, to at least one of the observables. We evaluated the PRCC considering normoxia and hypoxia scenarios (O2 = 209 *μM*or 9.9 *μM*, respectively), using 2300 runs (number of parameters evaluated ∗100), considering the sensitivity at time points 1h, 12h, 24h and 48h, in which we could see hypoxia effects clearly on the species while also including transient effects. We found that of the 23 parameters, 21 were highly influential (PRCC >0.2) to at least one of the observables across the selected time points.

Parameters were selected for calibration based on a combination of PRCC influence and condition-specific relevance to the calibration scenario. Specifically, parameters showing meaningful influence (PRCC >0.2) under hypoxia—the primary calibration condition—were prioritized for estimation. This yielded 15 parameters selected for calibration. One parameter, *delta_ox12*, showed moderate PRCC influence under normoxia (0.63) but low influence under hypoxia (0.29); despite passing the sensitivity-based collinearity screen, its inclusion in the optimization led to boundary convergence of multiple co-estimated parameters, indicative of nonlinear parameter interactions not captured by linear identifiability criteria. *delta_ox12* was therefore fixed at its estimated value and excluded from calibration. *Ko2* was included despite its moderate hypoxia influence (PRCC = 0.23) given its high normoxic influence (PRCC = 0.69) and its role in parametrizing the primary model input—oxygen concentration—which determines the normoxic steady state from which all simulations are initialized.

Figure 3C shows the PRCC results for each observable under both conditions. The 15 parameters selected for calibration were: *ktransc_base_mHIF1a*, *kdeg_mHIF1a*, *ktransc_base_mHIF2a*, *kactHtf*, *kdeg_TF*, *Ko2*, *Kd1*, *kdeghyp_mHIF1a*, *Kd2*, *ktransl_mHIF1*, *ktransl_mHIF2*, *kint*, *kp_mVEGFA*, *kform_phd2hif1*, and *delta_p3*. Non-identifiable unknown parameters (in SIA or estimability analysis) had their values fixed based on expected ranges reported in the literature. The complete parameter selection workflow, including the outcome of each filtering step and the rationale for exclusions at the calibration stage, is summarized in Table S9.

#### Global optimization and uncertainty quantification (UQ)

With the final set of fully identifiable unknown parameters, we performed global optimization to estimate their values by fitting the model to experimental data available in the literature. These data were represented by the observables, as previously defined. We separated the calibration into two steps, first calibrating the *HIF* mRNAs and then, as a second step, the remaining species (PHDs, HIF protein and *VEGFA* mRNA). We employed the global optimization algorithm Particle Swarm Optimization (PSO) available in SimBiology Model Analyzer, to search the parameter space and minimize the residuals between model predictions and experimental data. Prior to optimization, the model was run to steady state using the initial parameter guesses, and the steady-state values were used as the starting point for the optimization.

We performed a second round of local optimization using lsqnonlin on Simbiology Model Analyzer, for refining the most influential optimized parameters. Following optimization, we evaluated the goodness of fit using the Runs test, which computes the randomness of the distribution of the weighted sum of squared residuals between the model predictions and experimental data.^81^^,^^82^ The Runs test performed on the residuals returns two values: h (null-hypothesis rejection if = 1) and p (probability of observing a test statistic as extreme as (or more than) the value observed under the null hypothesis). In this test, high p-values represent residuals randomness, and low p-values represent residuals showing patterns. When the test did not reject the null hypothesis that residuals are in random order at p-value > 0.05, we considered the fit satisfactory, and we proceeded to evaluate the uncertainty of our predictions. In cases where the Runs test indicated non-random residuals, model fits were further inspected to determine whether deviations reflected systematic model deficiencies or localized discrepancies. If necessary, prediction bounds were adjusted and the global fitting procedure was repeated. However, when the model adequately captured the overall trends and amplitudes of the data, minor non-random patterns in the residuals were accepted and further analyses were performed.

After this step, uncertainty in the model predictions was quantified using a residual bootstrapping approach implemented in SimBiology (*sbiopredictionci*). We generated 1000 bootstrap replicates by resampling residuals from the locally-refined best-fit solution with replacement and adding them to the model-predicted outputs to create synthetic datasets. For each replicate, model predictions were recomputed, resulting in an empirical distribution of simulated values at each time point. The 2.5th and 97.5th percentiles of this distribution were then used to define the two-sided 95% prediction intervals, corresponding to the central 95% of the prediction distribution.

#### Validation

Finally, we performed model validation by comparing model-predicted time-courses against independent experimental datasets not used during calibration.^29^^,^^30^^,^^31^ Model performance was assessed using four complementary metrics: the raw coverage method, defined as the proportion of experimental data points falling within the 95% prediction interval over the full observation period^83^; the Runs test for randomness of prediction errors; the normalized root mean square error (NRMSE); and the Pearson correlation coefficient between predicted and observed values.

#### Modeled oxygen concentration

Oxygen concentration is the main factor to be considered in a model of hypoxia. A standard measure of O_2_ concentration is its partial pressure (pO_2_), which is different for every tissue and cell niche evaluated. It is usual to consider pO_2_ relative to atmospheric O_2_, expressed as a percent value. Considering that the atmospheric pressure at sea level is 760 mmHg, the partial pressure of O_2_ is 21% of that (∼160 mmHg). After accounting for water vapor pressure (∼47mmHg at body temperature), the pO_2_ is near 150mmHg. Traditionally, *in vitro* models consider 21% O_2_ as normoxia. It should be noted that when accounting for the 5% CO_2_ atmosphere typical of cell culture incubators, the actual O_2_ concentration in the incubator headspace is approximately 18.5%, however, we retain 21% O_2_ as our normoxic reference value for consistency with the reported values in experimental datasets used for model calibration and validation. While physiological pO_2_ levels to which ECs are exposed range from 1% to 15% O_2_, depending on tissue type and vessel location, *in vitro* models often consider 21% O_2_ a proxy for normoxia. This results in a physiologically inaccurate representation. This disparity has been recently discussed.^84^ Furthermore, recent evidence demonstrates that even controlled incubator setpoints may not accurately reflect the pericellular oxygen tension experienced by cells, as cellular oxygen consumption can substantially reduce local O_2_ levels below the nominal incubator value.^85^^,^^86^ *In vitro* models over the years have represented hypoxia with O_2_ levels at or below 1%.^11^^,^^18^^,^^27^^,^^29^ As most experimental models available in HUVECs *in vitro* use 21% or less as representative of normoxia and 1% or less as representative of hypoxia, these are the values we considered in our simulations. Although this has been discussed in the past, using these values allows us to observe changes in *HIF* and *VEGF* mRNA levels, therefore, they can be used as surrogates of hypoxia.

To convert O_2_ partial pressure (in mmHg) or % to a molar concentration (*μ*M) for use in a mechanistic computational model, we considered the oxygen solubility in water at 37°C to be ∼1.3 *μ*M/mmHg. Based on this, we estimated that 160 mmHg (≈21% O_2_) corresponded to ∼209 *μ*M and 7.6 mmHg (≈1% O_2_) corresponded to ∼9.9 *μ*M.

#### PHD activity inhibition simulations

To investigate model behavior under hypoxia in response to modulation of PHDs activities, we introduced scaling parameters representing the effective activity levels of PHD2 and PHD3 in the reactions governing HIF degradation. Specifically, the parameter *deact_PHD2hif1*, *deact_PHD2hif2* and *deactPHD3hif2* were incorporated as multiplicative factors in the corresponding PHD-mediated degradation terms for HIF1*α* and HIF2*α*.

These parameters represent the fraction of active enzyme, allowing simulation of partial or complete inhibition of PHD activity. Inhibition levels were implemented by reducing the values of these parameters from their baseline of 1 (no inhibition) to lower values corresponding to the desired degree of inhibition (e.g., 0.7, 0.4 and 0.1 for 30%, 60% and 90% inhibition, respectively). All simulations were performed under hypoxic conditions (1% O2).

### Quantification and statistical analysis

Model calibration was performed using Particle Swarm Optimization (PSO) followed by local refinement using lsqnonlin in SimBiology Model Analyzer (MathWorks, R2026a). Structural identifiability analysis was performed using GenSSI 2.0 and STRIKE-GOLDD 4.0. Practical identifiability analysis included local sensitivity analysis, collinearity analysis using cosine similarity, and partial rank correlation coefficient (PRCC) analysis.

Goodness-of-fit was evaluated using Pearson correlation coefficients, normalized root mean squared error (NRMSE), and Runs tests for residual randomness. Residual randomness was considered acceptable for p > 0.05. Uncertainty quantification was performed using residual bootstrapping with 1000 replicates to compute 95% prediction confidence intervals.

Sensitivity analyses were performed under normoxic (21% O_2_) and hypoxic (1% O_2_) conditions. PRCC analysis used 2300 runs and evaluated sensitivities at 1 h, 12 h, 24 h, and 48 h. Statistical details for each analysis are reported in the corresponding figures, figure legends, results, and supplementary tables.

## Supplemental information

Document S1. Figures S1–S4 and Tables S1–S7

Data S1. SBML implementation of the endothelial HIF signaling model, related to STAR Methods SBML file containing the mechanistic computational model of the PHD/HIF/VEGFA signaling pathway developed in this study.

Table S8. Dataset heterogeneity, related to STAR Methods Summary of all datasets used for model calibration and validation, including cell type, oxygen level, exposure duration, measurement platform, and normalization strategy.

Table S9. Parameter identifiability and calibration selection summary, related to STAR Methods Outcome of each filtering step in the parameter selection workflow, from structural identifiability analysis through final calibration selection, including rationale for exclusions.
