# Supplementary material for: A mechanistic computational model of the HIF signaling pathway in endothelial cells
Source: iScience. 2026 Jun 2;29(6):116195. doi: 10.1016/j.isci.2026.116195 (PMC13253090; doi:10.1016/j.isci.2026.116195)
Supplement: Document S1. Figures S1–S4 and Tables S1–S7 [file mmc1.pdf]

iScience, Volume *29*

Supplemental information

A mechanistic computational model of the HIF signaling pathway in endothelial cells

Rebeca Hannah de Melo Oliveira, Arvind P. Pathak, and Aleksander S. Popel

Supplementary Materials

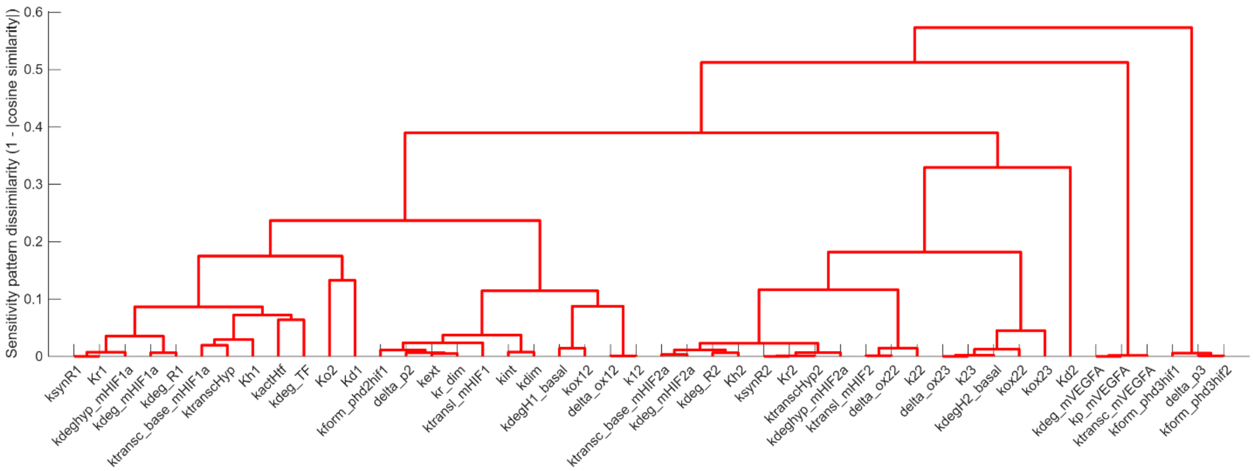

Fig S1. Hierarchical clustering of parameter sensitivity profiles

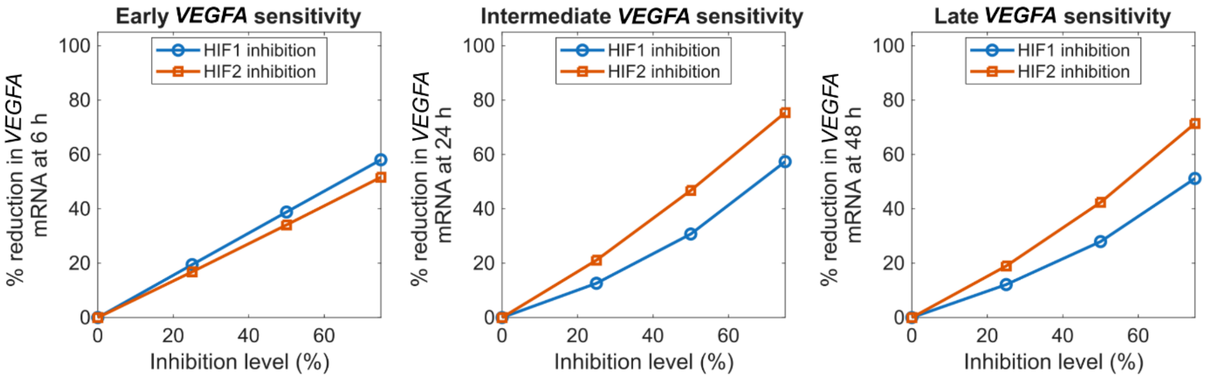

Fig S2: Time-dependent relative contributions of HIF1 and HIF2 proteins to *VEGFA* mRNA production. Percentage reduction in *VEGFA* mRNA at 6 h (early), 24 h (intermediate), and 48 h (late) following graded inhibition of HIF1α or HIF2α translation (0–75%) under hypoxia (1% O_2). At early time points, *VEGFA* mRNA is more sensitive to HIF1α inhibition, while HIF2α inhibition becomes progressively more impactful at later time points, reflecting the temporal shift in transcriptional control from HIF1α to HIF2α during sustained hypoxia.

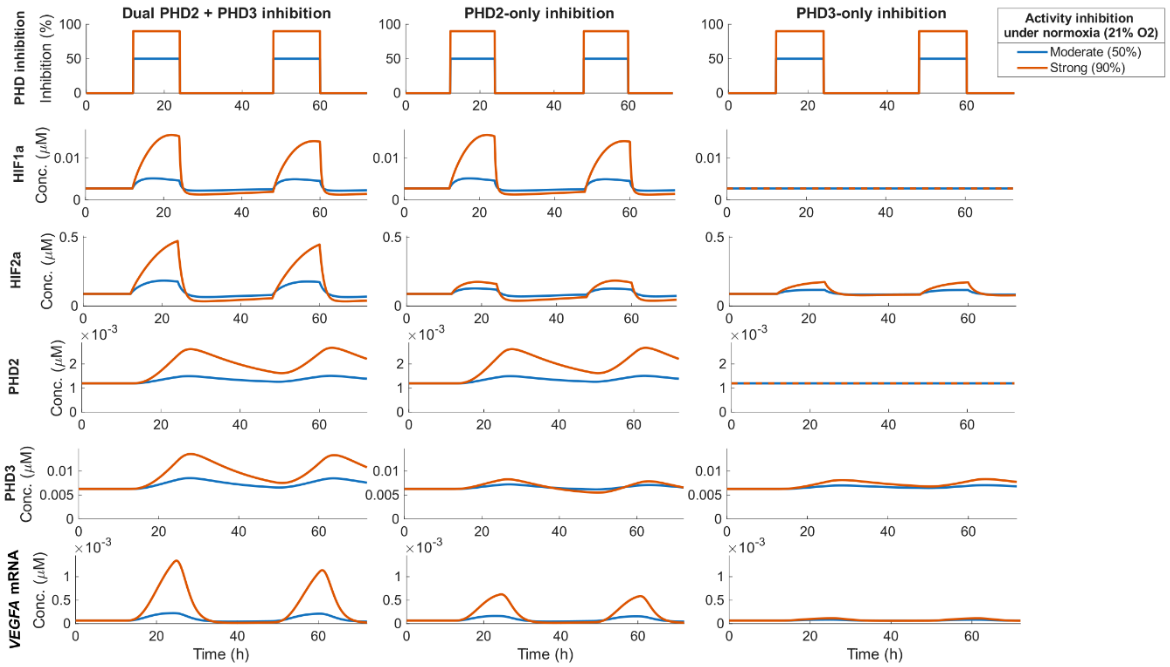

Fig S3: Cycles of PHD inhibition under normoxia conditions indicates isoform-specific effects. Simulated temporal profiles of HIF1α, HIF2α, PHD2, PHD3, and VEGFA mRNA under two cycles of PHD activity inhibition (12–24 h and 48–60 h) at moderate (50%, blue) and strong (90%, orange) inhibition levels under normoxic conditions (21% O_2). Results are shown for dual PHD2+PHD3 inhibition (left), PHD2-only inhibition (center), and PHD3-only inhibition (right).

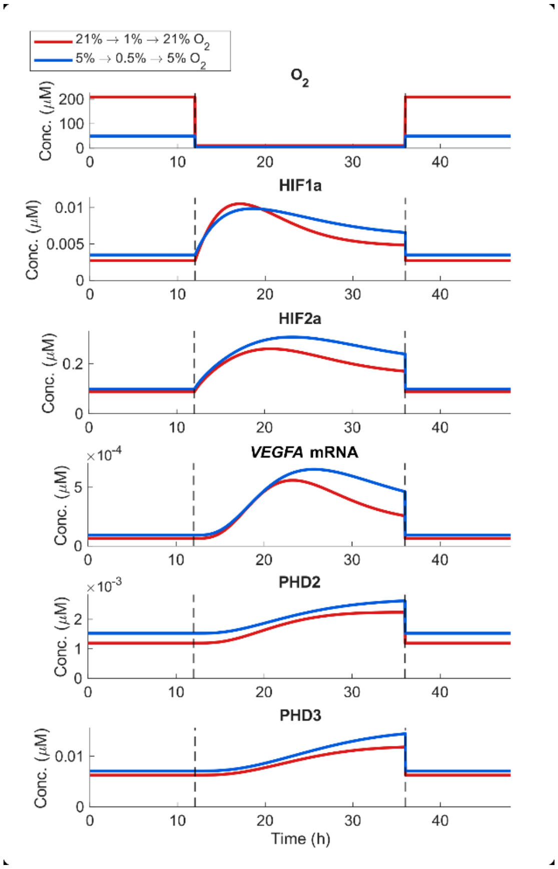

Fig S4: Simulated HIF and *VEGFA* mRNA responses under conventional and physioxic hypoxia-reoxygenation conditions. Simulated temporal profiles of O_2, HIF1α, HIF2α, *VEGFA* mRNA, PHD2, and PHD3 under a conventional in vitro transition (21% → 1% → 21% O_2, red) and a physioxic transition (5% → 0.5% → 5% O_2, blue), each initialized at their respective normoxic steady states. Dashed vertical lines indicate hypoxia onset and offset (12–36 h).

S1 Table: Species included in each model.

Model	HIFa protein	HIF mRNA	PHD	FIH	VEGF	CoCl2
Model 1*	HIF1a	Not included	PHD2	Included	VEGFA, VEGFA mRNA	Included
Model 2**	HIF1a, HIF2a	HIF1a mRNA, EPAS1	PHD2	Not included	Not included	Not included
Model 3***	HIF1a, HIF2a	HIF1a mRNA, EPAS1	PHD2, PHD3	Not included	Not included	Not included

* 1

** 2

*** 3

S2 Table: Model complexity

Model	Total # of species	Total # of parameters	Total # of reactions	Total # of ODEs
Model 1*	25	44	23	24
Model 2**	6	16	12	5

Model 3***	5	17	11	4

* 1

** 2

*** 3

S3 Table: Technical methodology

Model	Structural Identifiability	Practical Identifiability	Calibration Method	Validation / UQ	Sensitivity Analysis
Model 1*	Not included or reported	Not included or reported	Levenberg-Marquardt algorithm	Visual comparison	Local: complex-step approximation
Model 2**	Not included or reported ****	Not included or reported	Automatic fitting	Visual comparison	Not included or reported
Model 3***	Not included or reported	Not included or reported	Visual fitting	Visual comparison	Local

* 1

** 2

*** 3

**** Authors mention that the ODE model is under-determined, with many different values of parameters leading to similar results.

S4 Table: Parameters included in the model

**Name**	**Initial Value**	**Units**	**Interpretation**	**Source**
ktransc_base_mHIF1a	0.17	micromole/hour	Basal transcription rate of mHIF1α	Fit
kdeg_mHIF1a	0.17	1/hour	Degradation rate of mHIF1α mRNA	Fit
ktransc_base_mHIF2a	0.09	micromole/hour	Basal transcription rate of mHIF2α	Fit
kdeg_mHIF2a	0.087	1/hour	Degradation rate of mHIF2α mRNA	Fit
O2	209	micromole	Oxygen concentration under normoxia	Calculated
kactHtf	1	micromole/hour	Activation rate of HypoxiaTF by hypoxia	Assumed
kdeg_TF	1	1/hour	Degradation rate of HypoxiaTF	Assumed
Ko2	15	micromole	Oxygen sensitivity constant for HypoxiaTF activation	Assumed
ksynR1	1.084	1/hour	Synthesis rate of mHIF1α repressor	Fit
kdeg_R1	0.4	1/hour	Degradation rate of mHIF1α repressor	Fit
ksynR2	0.204	1/hour	Synthesis rate of mHIF2α regulator	Fit

kdeg_R2	0.05	1/hour	Degradation rate of mHIF2α regulator	Fit
ktranscHyp	0.35	micromole/hour	Hypoxia-induced transcription rate of mHIF1α	Fit
Kh1	0.05	micromole	Half-saturation constant for HypoxiaTF effect on mHIF1α	Fit
Kh2	0.15	micromole	Half-saturation constant for regulator effect on mHIF2α	Fit
Kr1	0.22	micromole	Repression constant for mHIF1α feedback inhibition	Fit
Kr2	0.15	micromole	Regulation constant for mHIF2α activation	Fit
Kd1	0.05	micromole	Hypoxia-dependent degradation constant for mHIF1α	Fit
Kd2	0.03	micromole	Hypoxia-dependent degradation constant for mHIF2α	Fit
kdeghyp_mHIF1a	0.313	1/hour	Hypoxia-induced degradation rate of mHIF1α	Fit
kdeghyp_mHIF2a	0.044	1/hour	Hypoxia-induced degradation rate of mHIF2α	Fit
ktranscHyp2	0.129	micromole/hour	Hypoxia-induced transcription rate of mHIF2α	Fit
kform_phd2hif1	0.3	1/hour	Production rate of PHD2 induced by HIF1	Fit
delta_p2	0.036	1/hour	Degradation rate of PHD2	Fit
kform_phd3hif1	0.025	1/hour	Production rate of PHD3 induced by HIF1	Fit
kform_phd3hif2	0.178	1/hour	Production rate of PHD3 induced by HIF2	Fit
delta_p3	0.8	1/hour	Degradation rate of PHD3	Fit

kdegH1_basal	0.07	1/hour	Basal degradation rate of HIF1α protein	Fit
kdegH2_basal	0.06	1/hour	Basal degradation rate of HIF2α protein	Fit
delta_ox12	100	1/hour	PHD2-mediated degradation rate of HIF1α	Fit
delta_ox22	100	1/hour	PHD2-mediated degradation rate of HIF2α	Fit
delta_ox23	300	1/hour	PHD3-mediated degradation rate of HIF2α	Fit
k12	0.05	micromole	Michaelis constant for PHD2–HIF1 interaction	Literature (Ferrante adapted)
k22	0.08	micromole	Michaelis constant for PHD2–HIF2 interaction	Literature (Ferrante adapted)
k23	5	micromole	Michaelis constant for PHD3–HIF2 interaction	Literature (Ferrante adapted)
kox12	100	micromole	Oxygen dependence constant for PHD2–HIF1 reaction	Literature
kox22	50	micromole	Oxygen dependence constant for PHD2–HIF2 reaction	Literature
kox23	15	micromole	Oxygen dependence constant for PHD3–HIF2 reaction	Literature
ktransl_mHIF1	0.01	1/hour	Translation rate of HIF1α protein	Fit
ktransl_mHIF2	0.04	1/hour	Translation rate of HIF2α protein	Fit
kint	1.105	1/hour	Internalization rate of HIF proteins	Fit
kext	1.08	1/hour	Recycling/export rate of HIF proteins	Fit
kdim	0.36	1/(micromole·hour)	Dimerization rate of HIF complexes	Fit
kr_dim	0.6	1/hour	Dissociation rate of HIF dimers	Fit

kdeg_mVEGFA	0.6	1/hour	Degradation rate of VEGFA mRNA	Fit
kp_mVEGFA	0.0906	micromole	Michaelis constant for VEGFA transcription	Literature (Zhao adapted)
ktransc_mVEGFA	0.5	micromole/hour	Transcription rate of VEGFA mRNA	Fit

S5 Table: List of model equations

Species	Equation
mHIF1a	(ktransc_base_mHIF1a) - (kdeghyp_mHIF1a*mHIF1a*(HypoxiaTF/(Kd1+HypoxiaTF))) + (ktranscHyp*(HypoxiaTF/(Kh1+HypoxiaTF))*(1/(1+(Rep_mHIF1a/Kr1)))) - (kdeg_mHIF1a*mHIF1a)
mHIF2a	(ktransc_base_mHIF2a) - (kdeg_mHIF2a*mHIF2a) + (ktranscHyp2*(HypoxiaTF/(Kh2+HypoxiaTF))*(1/(1+(Reg_mHIF2a/Kr2)))) - (kdeghyp_mHIF2a*mHIF2a*(HypoxiaTF/(Kd2+HypoxiaTF)))
Rep_mHIF1a	(ksynR1*HypoxiaTF) - (kdeg_R1*Rep_mHIF1a)
Reg_mHIF2a	(ksynR2*HypoxiaTF) - (kdeg_R2*Reg_mHIF2a)
HypoxiaTF	(kactHtf*(Ko2/(Ko2+O2))) - (kdeg_TF*HypoxiaTF)
PHD2	(kform_phd2hif1*HIF1ab) - (delta_p2*PHD2)
PHD3	(kform_phd3hif1*HIF1ab + kform_phd3hif2*HIF2ab) - (delta_p3*PHD3)
HIF1a	-(kdegH1_basal*HIF1a) + (ktransl_mHIF1*mHIF1a) - (kint*HIF1a - kext*HIF1a_n) - (deact_PHD2hif1*delta_ox12*O2/(O2+kox12)*HIF1a/(k12+HIF1a)*PHD2)
HIF2a	-(deact_PHD3hif2*delta_ox23*O2/(O2+kox23)*HIF2a/(k23+HIF2a)*PHD3) + (ktransl_mHIF2*mHIF2a) - (kint*HIF2a - kext*HIF2a_n) - (kdegH2_basal*HIF2a) - (deact_PHD2hif2*delta_ox22*O2/(O2+kox22)*HIF2a/(k22+HIF2a)*PHD2)
HIF1a_n	(kint*HIF1a - kext*HIF1a_n) - (kdim*HIF1a_n*HIF1b_n - kr_dim*HIF1ab)
HIF2a_n	(kint*HIF2a - kext*HIF2a_n) - (kdim*HIF2a_n*HIF1b_n - kr_dim*HIF2ab)
HIF1ab	(kdim*HIF1a_n*HIF1b_n - kr_dim*HIF1ab)
HIF2ab	(kdim*HIF2a_n*HIF1b_n - kr_dim*HIF2ab)
mVEGFA	-(kdeg_mVEGFA*mVEGFA) + (ktransc_mVEGFA*(HIF1ab/(HIF1ab+kp_mVEGFA))*(HIF2ab/(HIF2ab+kp_mVEGFA)))

S6 Table: List of model reactions

**Name**	**Reaction**	**Reaction Rate**
Basal transcription of mHIF1a	null -> mHIF1a	ktransc_base_mHIF1a
Hypoxia-induced degradation of mHIF1a	mHIF1a -> null	kdeghyp_mHIF1a*mHIF1a*(HypoxiaTF/(Kd1+HypoxiaTF))
Hypoxia-induced transcription of mHIF1a	null -> mHIF1a	ktranscHyp*(HypoxiaTF/(Kh1+HypoxiaTF))*(1/(1+(Rep_mHIF1a/Kr1)))
Basal degradation of mHIF1a	mHIF1a -> null	kdeg_mHIF1a*mHIF1a
Basal transcription of mHIF2a	null -> mHIF2a	ktransc_base_mHIF2a

Hypoxia-induced transcription of mHIF2a	null -> mHIF2a	ktranscHyp2*(HypoxiaTF/(Kh2+HypoxiaTF))*(1/(1+(Reg_mHIF2a/Kr2)))
Basal degradation of mHIF2a	mHIF2a -> null	kdeg_mHIF2a*mHIF2a
Hypoxia-induced degradation of mHIF2a	mHIF2a -> null	kdeghyp_mHIF2a*mHIF2a*(HypoxiaTF/(Kd2+HypoxiaTF))
Formation of HypoxiaTF	null -> HypoxiaTF	kactHtf*(Ko2/(Ko2+O2))
Degradation of HypoxiaTF	HypoxiaTF -> null	kdeg_TF*HypoxiaTF
Formation of mHIF1a repressor	null -> Rep_mHIF1a	ksynR1*HypoxiaTF
Degradation of mHIF1a repressor	Rep_mHIF1a -> null	kdeg_R1*Rep_mHIF1a
Formation of mHIF2a regulator	null -> Reg_mHIF2a	ksynR2*HypoxiaTF
Degradation of mHIF2a regulator	Reg_mHIF2a -> null	kdeg_R2*Reg_mHIF2a
HIF1-induced production of PHD2	null -> PHD2	kform_phd2hif1*HIF1ab
Degradation of PHD2	PHD2 -> null	delta_p2*PHD2
HIF-induced production of PHD3	null -> PHD3	kform_phd3hif1*HIF1ab + kform_phd3hif2*HIF2ab
Degradation of PHD3	PHD3 -> null	delta_p3*PHD3
Translation of HIF1a	null -> HIF1a	ktransl_mHIF1*mHIF1a
Basal degradation of HIF1a	HIF1a -> null	kdegH1_basal*HIF1a
PHD2-mediated degradation of HIF1a	HIF1a -> null	deact_PHD2hif1*delta_ox12*O2/(O2+kox12)*HIF1a/(k12+HIF1a)*PHD2
Nucleo-cytoplasmic shuttling of HIF1a	HIF1a <-> HIF1a_n	kint*HIF1a - kext*HIF1a_n
Translation of HIF2a	null -> HIF2a	ktransl_mHIF2*mHIF2a

Basal degradation of HIF2a	HIF2a -> null	kdegH2_basal*HIF2a
PHD2-mediated degradation of HIF2a	HIF2a -> null	deact_PHD2hif2*delta_ox22*O2/(O2+kox22)*HIF2a/(k22+HIF2a)*PHD2
PHD3-mediated degradation of HIF2a	HIF2a -> null	deact_PHD3hif2*delta_ox23*O2/(O2+kox23)*HIF2a/(k23+HIF2a)*PHD3
Nucleo-cytoplasmic shuttling of HIF2a	HIF2a <-> HIF2a_n	kint*HIF2a - kext*HIF2a_n
Dimerization of HIF1 complex	HIF1a_n + HIF1b_n <-> HIF1ab	kdim*HIF1a_n*HIF1b_n - kr_dim*HIF1ab
Dimerization of HIF2 complex	HIF2a_n + HIF1b_n <-> HIF2ab	kdim*HIF2a_n*HIF1b_n - kr_dim*HIF2ab
Transcription of mVEGFA	null -> mVEGFA	ktransc_mVEGFA*(HIF1ab/(HIF1ab+kp_mVEGFA))*(HIF2ab/(HIF2ab+kp_mVEGFA))

S7 Table: Calibrated parameters' values

Name	Global Calibration Value	Local Refinement Value	Unit
ktransc_base_mHIF1a	0.056000013	0.039875485	micromole/hour
kdeg_mHIF1a	0.372766733	0.50495796	1/hour
ktransc_base_mHIF2a	0.089464849	0.101065512	micromole/hour
kactHtf	1.294383083	1.083540036	micromole/hour
kdeg_TF	0.541777977	–	1/hour
Ko2	5.00000152	–	micromole
Kd1	0.14999983	–	micromole
kdeghyp_mHIF1a	0.070640194	–	1/hour
Kd2	0.015502206	–	micromole
ktransl_mHIF1	0.015539205	0.015539283	1/hour
ktransl_mHIF2	0.09	0.09	1/hour
kint	0.314455617	0.314453307	1/hour
kp_mVEGFA	0.03	0.03	micromole
kform_phd2hif1	0.9	–	1/hour
delta_p3	0.043640343	–	1/hour

Supplemental references

1. Ferrante, P., Preziosi, L., and Scianna, M. (2023). Modeling hypoxia-related inflammation scenarios. Math. Biosci. *355*, 108952. https://doi.org/10.1016/j.mbs.2022.108952.

2. Jaśkiewicz, M., Moszyńska, A., Króliczewski, J., Cabaj, A., Bartoszewska, S., Charzyńska, A., Gebert, M., Dąbrowski, M., Collawn, J.F., and Bartoszewski, R. (2022). The transition from HIF-1 to HIF-2 during prolonged hypoxia results from reactivation of PHDs and HIF1A

mRNA instability. Cell. Mol. Biol. Lett. *27*, 109–109. https://doi.org/10.1186/s11658-022-00408-7.

3. Zhao, C., and Popel, A.S. (2015). Computational Model of MicroRNA Control of HIF-VEGF Pathway: Insights into the Pathophysiology of Ischemic Vascular Disease and Cancer. PLOS Comput. Biol. *11*, e1004612. https://doi.org/10.1371/journal.pcbi.1004612.
